# Suicidality measured by PHQ-9 in kosovo during the COVID-19 outbreak

**DOI:** 10.1192/j.eurpsy.2021.272

**Published:** 2021-08-13

**Authors:** N. Fanaj, S. Mustafa, A. Ajradini, B. Kabashaj, I. Poniku

**Affiliations:** 1 Qshm, Mental Health Center Prizren, PRIZREN, Kosovo; 2 Sociology, University of Prishtina, Prishtina, Kosovo; 3 Psychiatry, Regional Hospital, PRIZREN, Kosovo

**Keywords:** COVID-19, Suicide, Kosovo, PHQ-9

## Abstract

**Introduction:**

As a consequence of the impact of COVID-19 there are suggestions and projections that suicide rates will rise, although this is not inevitable. It is intriguing what impact it will have on Kosovo as a country with the lowest suicide rate in Europe.

**Objectives:**

The objective of this study was to understand the level of suicidal thinking as a result of the COVID-19 situation and possible associations with sociodemographic variables.

**Methods:**

It’s a comparative study. We examined data of two cross-sectional online surveys conducted during the one-month periods 20.03.20 until 23.04.20 and 27.04.2020 until 05.06.2020. The participants were online respondents, N = 194 (first period) and N = 155 (second period); who completed the Albanian version of PHQ-9. We used the statement number 9 of questionnaire indicative of suicide.

**Results:**

Mean score of suicidal thinking resulted 0.58 (SD = .98) in the first period and 0.84 (SD = 1.16) in the second period. 10.1% of participants in March/April and 18.2% in May/June period scored that almost every day thoughts that would be better off dead, or of hurting yourself in some way. Significantly higher suicidal thinking resulted for females and for respondents who had previously had depression in both periods. Compared to two previous studies (2018 and 2019) in different settings only the results of the second period show an increase in suicidal thinking.

**Conclusions:**

Further studies are needed to better scientifically elaborate these findings. It is important enhanced surveillance of COVID-19-related risk factors contributing to suicidal behaviors and timely preventive efforts.

**Disclosure:**

No significant relationships.

